# Review of range‐wide vital rates quantifies eastern wild Turkey population trajectory

**DOI:** 10.1002/ece3.9830

**Published:** 2023-02-22

**Authors:** David W. Londe, Anna K. Moeller, Paul M. Lukacs, Samuel D. Fuhlendorf, Craig A. Davis, Robert Dwayne Elmore, M. Colter Chitwood

**Affiliations:** ^1^ 008c Ag Hall, Department of Natural Resources Ecology and Management Oklahoma State University Stillwater Oklahoma USA; ^2^ Wildlife Biology Program, Department of Ecosystem and Conservation Sciences, W.A. Franke College of Forestry and Conservation University of Montana Missoula Montana USA

**Keywords:** eastern wild turkey, elasticity, life‐stage simulation analysis, *Meleagris gallopavo silvestris*, population growth, survival

## Abstract

Recent declines in eastern wild turkeys (*Meleagris gallopavo silvestris*) have prompted increased interest in management and research of this important game species. However, the mechanisms underlying these declines are unclear, leaving uncertainty in how best to manage this species. Foundational to effective management of wildlife species is understanding the biotic and abiotic factors that influence demographic parameters and the contribution of vital rates to population growth. Our objectives for this study were to (1) conduct a literature review to collect all published vital rates for eastern wild turkey over the last 50 years, (2) perform a scoping review of the biotic and abiotic factors that have been studied relative to wild turkey vital rates and highlight areas that require additional research, and (3) use the published vital rates to populate a life‐stage simulation analysis (LSA) and identify the vital rates that make the greatest contribution to population growth. Based on published vital rates for eastern wild turkey, we estimated a mean asymptotic population growth rate (*λ*) of 0.91 (95% CI = 0.71, 1.12). Vital rates associated with after‐second‐year (ASY) females were most influential in determining population growth. Survival of ASY females had the greatest elasticity (0.53), while reproduction of ASY females had lower elasticity (0.21), but high process variance, causing it to explain a greater proportion of variance in *λ*. Our scoping review found that most research has focused on the effects of habitat characteristics at nest sites and the direct effects of harvest on adult survival, while research on topics such as disease, weather, predators, or anthropogenic activity on vital rates has received less attention. We recommend that future research take a more mechanistic approach to understanding variation in wild turkey vital rates as this will assist managers in determining the most appropriate management approach.

## Graphical abstract

Using a life‐stage simulation analysis, we show survival and reproduction of adult female wild turkeys had the greatest influence on population trajectories for this species. However, these life stages showed differing patterns in elasticity and variability, with adult survival having high elasticity but low process variance, and reproduction having lower elasticities but greater process variance. Additionally, we highlight several critical knowledge gaps regarding vital rates for different life stages and in the factors regulating or limiting wild turkeys.

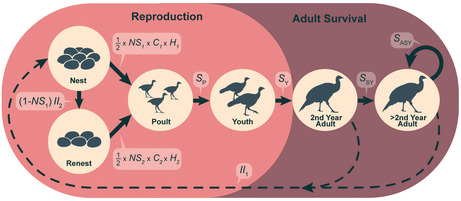

## 1 INTRODUCTION

Eastern wild turkeys (*Meleagris gallopavo silvestris*; hereafter, wild turkey) are a widespread and abundant gamebird species that inhabit a variety of landscapes in eastern North America. Overhunting and habitat loss in the early 1900s resulted in the extirpation of the wild turkey from much of its distribution (Baily, 1980), but extensive restoration and translocation efforts led to wild turkey populations not only recovering but also greatly expanding outside of their historical distribution in recent decades (Eriksen et al., 2015). Following successful restoration, many states liberalized wild turkey hunting regulations (Isabelle et al., 2018), making this species economically important as well (Chapagain et al., 2020). However, wild turkey populations have begun to decline again throughout the United States (Casalena et al., 2015; Eriksen et al., 2015), with many states reporting reduced poult‐to‐hen ratios, suggesting changes in productivity (Byrne et al., 2015). The mechanisms underlying these declines remain unclear in many locations and may be the result of several potential factors throughout the wild turkey's distribution including changes in habitat, weather, or predator communities or increased disease prevalence (Casalena et al., 2015, Eriksen et al., 2015). The widespread nature of these declines has prompted increased interest and investment in research to identify the causative factors and determine the best management strategies to stabilize wild turkey populations.

Understanding the contribution of different life history stages or vital rates to a population's growth rate is a fundamental goal of population ecology, as this knowledge can be used to identify the life stages that can be targeted most effectively for management (Crowder et al., 1994; Johnson et al., 2010; Mills & Lindberg, 2002). As part of this, it is necessary to consider the natural range of variability for vital rates, as small changes in some vital rates may cause substantial changes in population growth (i.e., high elasticity) while also exhibiting relatively little variation in wild populations, leaving few opportunities to alter these vital rates through management (Gaillard et al., 1998; Mills et al., 1999). Alternatively, vital rates that have relatively small influences on population growth (i.e., low elasticity) may have greater effects on population size if these vital rates also exhibit high levels of variability within and between populations (Chitwood et al., 2015; Coulson et al., 2005; Raithel et al., 2007). The use of life‐stage simulation analysis (LSA; Wisdom et al., 2000) has been especially valuable for the identification of vital rates that have the greatest impact on population growth rate. This is because LSA allows for the modeling of population growth or persistence using complex age and life history structures while incorporating information about variability in vital rates into a single framework (Wisdom et al., 2000). Further, the results from these models can serve as the foundation for subsequent simulations to evaluate how management actions that change vital rates may affect population growth (Mills et al., 1999).

An important challenge associated with studying wildlife populations is assessing the biotic and abiotic factors that influence population demographics and understanding how much actual control managers may have in altering specific vital rates. Many wildlife populations may undergo substantial year‐to‐year variation because of weather, disease, predation, or interspecific/intraspecific competition (Sibly & Hone, 2002). For harvested wildlife populations, hunting season length, timing, and harvest rate can have significant impacts on subsequent population sizes as well (Cooch et al., 2014; Ginsberg & Milner‐Gulland, 1994). However, the importance of different factors in determining vital rates often varies temporally, spatially, and with population size (i.e., density‐dependent factors), complicating the process of determining the mechanisms underlying population variability for many wildlife populations (Krebs, 2002). For some species, the importance of different factors in regulating or limiting population growth has been the source of intense debate (Martínez‐Padilla et al., 2014). Lack of certainty about the factors most important to influencing a species growth rate can place a limit on a manager's ability to address population declines (Runge et al., 2011) or lead to ineffective or counterproductive management practices and reduce public trust in management agencies (Riley et al., 2018).

Wild turkeys have been the subject of considerable research over the last 50 years, resulting in a large body of literature. A synthesis of wild turkey vital rates and the factors that influence them across their distribution could provide a clearer understanding of potential causes for the recent large‐scale decline in wild turkeys. Therefore, the objectives of this study were to (1) conduct a review of published wild turkey literature over the last 50 years to obtain vital rates across the distribution of eastern wild turkey, (2) perform a scoping review of the biotic and abiotic factors that have been studied in relation to wild turkey vital rates, and (3) use the published vital rates to populate an LSA population model and identify the life stages that provide the greatest contribution to wild turkey population growth rate and identify research needs for wild turkey demographic data. Our scoping review specifically focused on population studies that report vital rates and the factors that alter wild turkey demographics. As a result, our goal was to review the existing literature to highlight trends and patterns in wild turkey research as well as highlight gaps in our knowledge of the factors that influence wild turkey vital rates. Using both quantitative methods (LSA) and a qualitative review (scoping review), our goal was to utilize existing research to improve our understanding of wild turkey population dynamics and to help inform discussions regarding wild turkey research and management.

## 2 METHODS

### 2.1 Literature review

In June 2021, we used SCOPUS and Google Scholar to conduct a web search of all ecological and wildlife journals to locate peer‐reviewed articles that reported vital rates for wild turkeys (eastern subspecies only). We used combinations of primary search terms (i.e., wild turkey, eastern wild turkey, *Meleagris gallopavo silvestris*, *Meleagris gallopavo*) and secondary search terms (i.e., survival, adult survival, nest success, poult survival, recruitment, clutch size, vital rates, and demographic rates) to develop a list of titles and abstracts for publications that reported information about wild turkey vital rates. We also searched the literature‐cited sections of published articles for additional publications. We excluded government reports and unpublished theses and dissertations from our final list because it was unclear to what extent most of these documents had undergone peer review and because much of the information from these sources could be found in peer‐reviewed outlets gathered by our search. Our search process also yielded papers focused on other wild turkey subspecies (e.g., Merriam's [*M. gallopavo merriami*], Rio Grande [*M. gallopavo intermedia*], and Gould's [*M. gallopavo mexicana*]). Additionally, we conducted a complete review of the Proceedings of the National Wild Turkey Symposium (1959–2016), following the same procedure described below to extract vital rates and information about factors that influence wild turkey demography.

**FIGURE 1:**
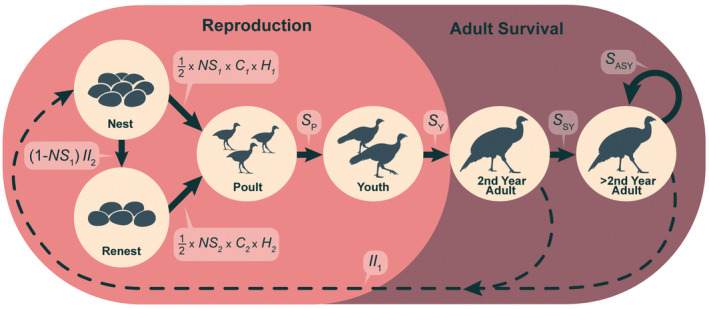
Conceptual model of eastern wild turkey life history stages used to parameterize the life‐stage simulation analysis. Life stages indicated by circles with bold arrows showing the transitions to subsequent life stages. Vital rates associated with each life‐stage transition indicated by italicized parameters and equations. Vital rates associated with reproduction include incubation initiation (II), nest success (NS), clutch size (C), hatching rate (H), poult survival (S_P_), and youth survival (*S*_Y_). Vital rates associated with the adult stages include second‐year adult survival (*S*_SY_) and after‐second‐year adult survival (*S*_ASY_).

From the journal articles and conference proceedings retained for further review, a single reviewer (DWL) examined each paper and extracted any vital rates that were reported for males or females (vital rates defined in Appendix 1; Figure 1), as well as associated sample sizes and error estimates (e.g., standard errors, standard deviations, and confidence intervals). When no error estimates or sample sizes were reported, we still recorded the vital rate, but those entries were only used for summary statistics and not in the subsequent distribution‐wide analysis. In addition to vital rates, we assessed each paper to determine whether it evaluated possible mechanisms for variation in vital rates. We considered a paper to have evaluated a mechanism if it reported some causative or correlative statistical analysis between a vital rate and an abiotic or biotic variable. Importantly, we did not consider hypotheses introduced by the authors in the introduction or discussion as a possible mechanism if the paper did not also include a quantitative evaluation of that mechanism (e.g., Wright et al. (1996) suggested low overwinter survival was the result of above average snowfall but did not provide an analysis to support the statement). As we only reviewed studies that reported vital rates, we did not include studies on other topics such as behavior, habitat use, or disease occurrence. These studies are important, as they provide insight into wild turkey ecology and management, but because they do not provide a direct evaluation of how these factors influence vital rates they were not included in our review.

For studies that evaluated possible mechanisms for variation in vital rates, we categorized each of the possible mechanisms into five broad categories that described either intrinsic or extrinsic factors that may influence wild turkey populations. Within each of the five broad categories, we further classified studies into finer‐scale subcategories. These categories and subcategories were selected because they represented different groups of variables that are believed to influence gamebird species population dynamics and were proposed in a conceptual model by Weinstein et al. (2007) to be important in influencing wild turkey populations. For intrinsic factors, we included a category for individual or behavioral factors that included subcategories for age, experience or body condition, movement/space use, social structure, genetics, or life stage/behavioral state. For extrinsic factors, we included categories for biotic interactions (e.g., predation, disease/parasitism, and inter/intraspecific competition), habitat factors (e.g., fine‐scale habitat, landscape‐scale habitat, habitat management, and forage availability/quality), weather conditions (e.g., breeding season weather and nonbreeding season weather), and anthropogenic factors (e.g., direct effects of harvest, indirect effects of harvest, and nonhunting‐related human‐related factors). We summarized the number of studies in each of these categories to provide an overview of existing published work and to highlight potential gaps in the literature.

### 2.2 Data analysis

Using the vital rates extracted from the literature, we conducted a life‐stage simulation analysis (LSA; Wisdom et al., 2000) for eastern wild turkey. Wild turkey populations have expanded considerably beyond their historic distribution (Eriksen et al., 2015), but we restricted our LSA to vital rates collected within the eastern wild turkey's historic distribution, as vital rates from newly colonized areas may not be representative of population dynamics within the historic distribution. We defined a female‐only prebirth pulse matrix model for two stages (SY = second‐year individuals [i.e., individuals approximately 1‐year‐old], ASY = after‐second‐year adults [i.e., individuals approximately 2+ years old]):

$$\left[\begin{array}{cc} R_{\mathrm{SY}} & R_{\mathrm{ASY}}\\ S_{\mathrm{SY}} & S_{A\mathrm{SY}} \end{array}\right],$$

where *R* represents annual reproductive output and *S* represents annual survival rate. We limited our model to two adult stage classes because of the difficulty of correctly aging adult wild turkeys in the field beyond these broad classes. We defined second‐year individuals (SY) as birds captured in the winter/spring prior to their first breeding season. These birds were typically between 9 and 10 months of age at capture and may be variously described as juveniles, yearlings, or subadults in the literature. We defined after‐second‐year (ASY) adults as individuals that were known to be >1‐year‐old and entering into at least their second breeding season (but their exact age was unknown). We did not include data from studies that did not report stage‐specific vital rates in our subsequent models.

We defined *R* from the following components (see Equation 2): incubation initiation (II), apparent nest success (NS), clutch size (*C*), hatching rate (*H*), apparent poult survival to 28 days (PS), and youth survival from 29 to 365 days (*S*_Y_; Figure 1; McCaffery & Lukacs, 2016; Taylor et al., 2012). Full vital rate definitions and how they were calculated can be found in Appendix 1. No studies reported survival for the youth period, and as a result, we estimated this vital rate based on survival estimates from SY individuals following methods from previous studies reporting population models for wild turkeys (Lehman et al., 2022; Pollentier et al., 2014; Roberts & Porter, 1996; Rolley et al., 1998). We estimated youth survival from 29–365 days (*S*_Y_) from second‐year individual annual survival estimates standardized to this shorter time period, using

(1) $$S_{Y}={S_{\mathrm{SY}}}^{\left(365-28\right)/365}.$$

While we recognize this assumption likely overestimates survival during this period, as youth survival is typically lower than adult survival for game bird species (Taylor et al., 2012), we used this approach because it allowed us to remain consistent with existing methods for wild turkey population models, and it provided a baseline estimate for this period. More research is needed to quantify survival estimates for this period. Additionally, nest initiation is typically reported as the proportion of hens that began incubating a nest; however, these estimates likely do not accurately reflect nesting efforts, as hens that failed to begin incubation may have attempted a nest but lost it prior to the onset of incubation (Blomberg et al., 2015; McPherson et al., 2003). To highlight this potential bias in reported vital rates, we refer to nest initiation rates as incubation initiation (II).

We allowed reproductive vital rates to vary between nesting attempts and by the stage class of the female (second year individual, after‐second‐year adult). Wild turkeys generally only attempt a second nest if the first nest fails (1−NS_1_), so a single hen's reproductive contribution for a year can come from either a first nest or a second nest (Vangilder & Kurzejeski, 1995). Although additional nest attempts after failure of the second nest may occur, these nesting attempts make up a small proportion of overall nest attempts (Keever et al., 2022), so we did not include them in our model. Therefore, we defined reproduction for each stage class (*a*) as:

(2) $$\begin{array}{cc} R_{a}= & \left[{\mathrm{II}}_{1a}\times {\mathrm{NS}}_{1a}\times \frac{C_{1a}}{2}\times H_{1a}\times {\mathrm{PS}}_{a}\times S_{Y}\right]\\ & +\left[{\mathrm{II}}_{1a}\times \left(1-{\mathrm{NS}}_{1a}\right)\times {\mathrm{II}}_{2a}\times {\mathrm{NS}}_{2a}\times \frac{C_{2a}}{2}\times H_{2a}\times {\mathrm{PS}}_{a}\times S_{Y}\right] \end{array}$$

The additive (bracketed) terms in the equation represent the number of poults from a first or second nesting attempt, respectively, that survive to 1 year of age. We assumed an equal sex ratio of eggs and therefore divided clutch size by 2 to estimate the number of female poults. While previous studies have indicated the potential for a male bias in brood sex ratios in the Rio Grande subspecies of wild turkeys (Collier et al., 2007), similar patterns have yet to be described in other wild turkey subspecies.

Because vital rate data were reported differently across studies, some standardization was necessary for use in our analysis. First, we transformed all vital rate estimates reported as percentages to probabilities by dividing the estimate and its standard error by 100. Second, we transformed mortality to survival by subtracting the mortality estimate from 1 (standard error remained unchanged under the binomial distribution). Third, we removed duplicate estimates to the best of our ability. For example, one study (Shields & Flake, 2006) reported apparent poult survival from 0–14, 14–28, and 0–28 days (inclusive of the previous two estimates), so we only used the estimate for 0–28 days.

After standardizing the vital rates, we removed estimates that combined SY and ASY wild turkeys or did not report standard error or another measure of variation that allowed us to estimate standard error. For estimates that did not report a standard error but included another measure of variation, we calculated standard error in one of three ways. First, for estimates that reported a standard deviation and assumed a normal sampling distribution of the vital rate, we calculated standard error by dividing the standard deviation by the sample size. Second, for estimates that reported a confidence interval but no standard error, we assumed the sampling distribution was normally distributed and calculated SE from the CI as: (upper limit – lower limit)/3.92 for 95% CI, and (upper limit – lower limit)/3.29 for 90% CI. Third, for estimates of nesting rate, apparent nest success, or survival that had no reported SE or CI but reported sample size, we estimated standard error from the binomial distribution as:

(3) $$\mathrm{SE}=\sqrt{\frac{p\left(1-p\right)}{n}},$$

where *p* represents the point estimate of the vital rate and *n* represents the sample size. If we were not able to estimate standard error in any of these ways or the reported standard error was 0, we removed the estimate from our analysis.

To complete the LSA, we created a process distribution for each vital rate defined in our matrix. The process distribution is a curve that describes the variability of a vital rate across populations and years. When measuring vital rates, researchers observe only a realization of the vital rate, and reported measures of variance (e.g., confidence intervals) encompass both biological process variance (true variance in a vital rate resulting from spatial or temporal variation in habitat, population dynamics, or life history) and variation resulting from sampling error (Raithel et al., 2007; White, 2000). To correctly build the process distribution, variation from sampling error must be separated from the process variance (White, 2000). To address this challenge, we used a Bayesian modeling approach, which improves upon the Method of Moments approach proposed by White (2000) by directly estimating a posterior distribution, which is equivalent to the process distribution that is desired. To estimate the process distribution without sampling error, we modeled the observed estimates as random variables drawn from a normal distribution centered on the true parameter value, with a standard deviation equal to the standard error of the estimate. For example, we used observations of incubation initiation probability (*y*_II_) for age class *a* and nest attempt *n* to estimate mean incubation initiation probability II via the equation:

(4) $$y_{{\mathrm{II}}_{a,n}}∼\text{Normal}\left({\mathrm{II}}_{a,n},\mathrm{SE}\left(y_{{\mathrm{II}}_{a,n}}\right)\right).$$

We did not have data on hatching rates for renesting attempts because few studies reported it separately for adult age classes, so we estimated the process distribution of hatching rate from first nests only.

We ran models for all parameters in JAGS 4.3.0 (Plummer, 2003). We used flat Uniform (0, 1) priors for all vital rates except clutch size, which we gave a normal prior centered on the mean observed clutch size, with a standard deviation equal to the standard deviation of observed clutch sizes. We chose to use truncated normal distributions in the LSA simulation because our process distributions were approximately normal and centered so that approximately 100% of the distribution was between 0 and 1. We ran three chains for 30,000 iterations with the first 10,000 as burn‐in, with no thinning. We inspected the MCMC plots visually for convergence and checked for R‐hat values <1.1 (Gelman & Rubin, 1992). The mean and standard deviation of the posterior distribution describe the process distribution of each vital rate (Table 1).

**TABLE 1:** Range‐wide process distributions and number of vital rate estimates (*n*) used for second‐year (SY) and after‐second‐year (ASY) adult eastern wild turkey in life‐stage simulation analysis.

Vital rate	Age of female	Nest attempt	Mean	Standard deviation	2.5%	97.5%	*n*
Clutch Size	SY	1	10.72	0.28	10.16	11.27	6
Clutch Size	ASY	1	10.91	0.27	10.37	11.44	6
Clutch Size	SY	2	10.14	0.54	9.08	11.2	1
Clutch Size^a^	ASY	2	10.68	1.18	8.36	12.99	0
Hatching Rate	SY	1	0.83	0.1	0.62	0.99	4
Hatching Rate	ASY	1	0.83	0.09	0.64	0.98	4
Nest Initiation	SY	1	0.73	0.06	0.61	0.86	17
Nest Initiation	ASY	1	0.87	0.05	0.77	0.96	18
Nest Initiation	SY	2	0.26	0.08	0.11	0.41	14
Nest Initiation	ASY	2	0.25	0.06	0.12	0.38	14
Nest Success	SY	1	0.29	0.07	0.14	0.43	15
Nest Success	ASY	1	0.38	0.06	0.27	0.49	24
Nest Success	SY	2	0.44	0.21	0.06	0.87	4
Nest Success	ASY	2	0.23	0.11	0.04	0.45	5
Poult Survival	SY		0.24	0.13	0.02	0.51	3
Poult Survival	ASY		0.33	0.12	0.09	0.57	3
Reproduction^b^	SY		0.18	0.11	0.02	0.43	NA
Reproduction^b^	ASY		0.34	0.15	0.09	0.66	NA
Annual Survival	SY		0.6	0.05	0.5	0.7	18
Annual Survival	ASY		0.64	0.05	0.54	0.74	18
Youth Survival (28–365 days)^c^			0.63	0.05	0.53	0.72	18

*Note*: Ninety‐five percent of process variation is bounded by lower (2.5%) and upper (97.5%) percentiles. Process distributions based on data extracted from eastern wild turkey vital rates published between 1970 and 2021.

Abbreviations: ASY, After‐second‐year adult; SY, Second year individual.

^a^ Sample size of 0 indicates estimate equivalent to prior.

^b^ Reproduction estimated from Equation (1); value derived, so no sample size.

^c^ No studies reported youth survival; estimated based on second‐year adult survival following Roberts and Porter (1996).

After defining the process distributions for our vital rates, we performed the LSA in R 4.1.3 (R Core Team, 2022). For each of 10,000 replicates, we drew a value for each vital rate from either a normal distribution (for clutch size) or a truncated normal defined between 0 and 1 (for all other vital rates). We used Equation (2) to calculate reproduction for each replicate and populated our matrix model accordingly. We assumed no correlation structure among vital rates because few estimates exist for these parameters in wild turkeys (Alpizar‐Jara et al., 2001). We calculated the asymptotic growth rate, *λ*, from the dominant eigenvalue for each simulation replicate. We calculated elasticity for each replicate using the R package popbio and calculated mean elasticities across all replicates (Stubben & Milligan, 2007). Finally, we performed linear regressions to compare our 10,000 values of *λ* to the 10,000 values of each vital rate. We used the resulting coefficient of determination (*R* ^2^) values to determine the amount to which variation in each vital rate explained variation in *λ* (Wisdom et al., 2000).

## 3 RESULTS

**FIGURE 2:**
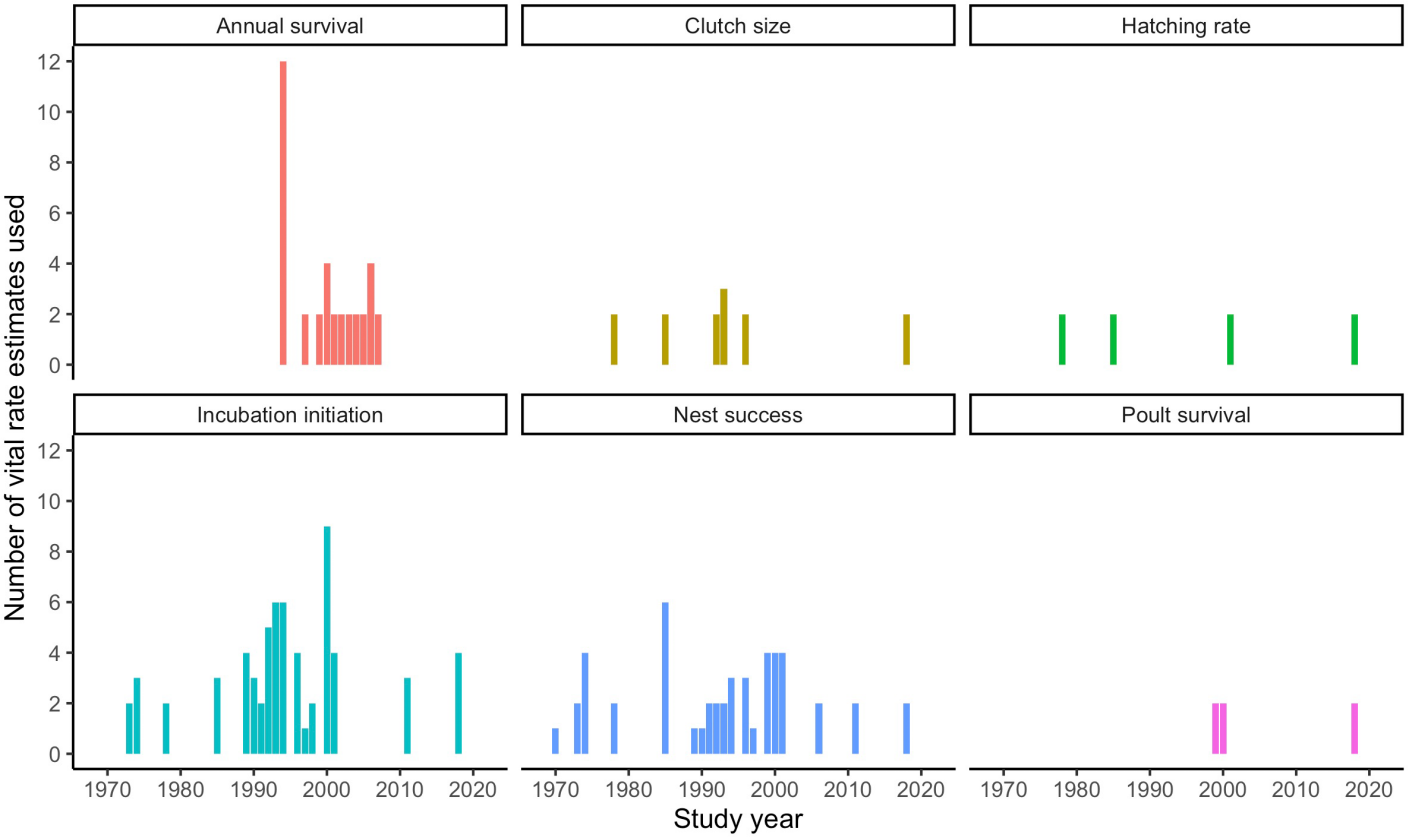
Number of eastern wild turkey vital rate estimates used in the life‐stage simulation analysis by the year they were published (1970–2021). We used only vital rates that were estimated for second‐year or after‐second‐year adult female eastern wild turkeys and included a measure of variation that could be converted into standard error. If the estimate was an average over a range of years, we plotted the estimate in the final year. If no year was associated with the estimate, we used the publication year. We show all estimates of clutch size, hatching rate, apparent nest success, apparent poult survival, and incubation initiation, regardless of nesting attempt.

Our literature review resulted in an initial list of 89 peer‐reviewed journal articles that reported vital rates for wild turkey. Twenty‐one (24%) were focused on subspecies other than eastern wild turkey and were excluded from subsequent analyses. This left 68 papers (76%) for analysis inclusion, including 20 from the National Wild Turkey Symposia and 48 from peer‐reviewed journals. Publication dates ranged from 1970 to 2021 (Figure 2). The most widely reported vital rate was apparent nest success (*n* = 36; 53%), followed by incubation initiation (*n* = 31; 45%), annual survival (*n* = 28; 41%), and apparent poult survival (*n* = 20; 29%). Notably, no studies (0%) reported youth survival (i.e., survival from 28 days to the first breeding season). Of these 68 papers retained for analysis, 45% did not evaluate any underlying mechanisms (e.g., weather variability, predator populations, and habitat) for variation in vital rates and only presented vital rate estimates and raw sources of mortality.

### 3.1 Scoping review‐‐nests

The most studied factor relating to nest survival was the effects of vegetation, cover, or habitat (*n* = 17; 25%; Table 2). Studies occurred at both fine scale (i.e., vegetation composition or structure at the nest site) and landscape scale (i.e., composition of habitat over large areas or distance to landscape features), with nine studies (13%) and eight studies (12%), respectively (Table 2). Only three studies (4%) directly evaluated the effects of habitat management on nest sites, with all three studies being related to prescribed fire. One study (1%) examined the effects of predator removal. Nine studies (10%) evaluated intrinsic factors that may influence apparent nest success, with five (7%) of those studies evaluating the effects of the attending hen's age or body condition and the remaining three (4%) studies evaluating the effects of the attending hen's space use on apparent nest success (Table 2). Four studies (5%) reported effects of weather on apparent nest success, and no studies (0%) reported effects of biotic interactions (e.g., changes in predator communities or densities) for apparent nest success, despite predation being frequently reported as the main source of nest loss.

**TABLE 2:** Summary of the number of studies evaluating how biotic and abiotic factors influence life‐stage vital rates for eastern wild turkeys from 1970 to 2021.

Categories and subcategories	Nesting	Poult survival	Youth survival	Adult survival
Individual/behavioral factors				
Age/experience/body condition	6^3,25,28,31,37,39^	1^38^		7^9,10,19,21,26,33,40^
Genetics				
Movement and space use	4^1,2,3,18^	1^6^		1^12^
Reproductive status/seasonal variation				4^4,17,19,21^
Biotic interactions				
Predation	1^29^			
Competition (inter or intraspecific)				
Disease/parasitism				
Habitat				
Fine scale habitat	9^3,11,14,16,18,36,38,41,42^	2^22,41^		
Landscape scale habitat	8^8,11,14,16,36,38,41,42^	2^22,41^		3^12,24,32^
Forage availability				
Weather				
Breeding season weather	5^15,19,20,35,38^	3^34,38^		
Nonbreeding season weather	1^15^			1^15^
Anthropogenic factors				
Hunting season timing and duration				6^5,7,9,23,26,27^
Habitat management	3^14,30,42^			2^13,24^

*Note*: We only included studies that contained either causal or correlative statistical analysis between a vital rate and an abiotic or biotic variable. Blank cells indicate no studies were identified for that topic and life stage.

^1^Bakner et al. (2019), ^2^Badyaev and Faust (1996), ^3^Badyaev et al. (1996), ^4^Byrne and Chamberlain (2018), ^5^Chamberlain et al. (2012), ^6^Chamberlain et al. (2020), ^7^Conner et al. (2006), ^8^Crawford et al. (2021), ^9^Diefenbach et al. (2012), ^10^Eriksen et al. (2010), ^11^Fuller et al. (2013), ^12^Hubbard et al. (1999a), ^13^Kane et al. (2007), ^14^Kilburg et al. (2014), ^15^Lavoie et al. (2017), ^16^Little et al. (2014), ^17^Little et al. (2016), ^18^Lohr et al. (2020), ^19^Lowrey et al. (2000), ^20^Miller, Leopold, and Hurst (1998), ^21^Miller, Burger, et al. (1998), ^22^Metzler and Speake (1985), ^23^Moore et al. (1993), ^24^Niedzielski and Bowman (2015), ^25^Norman et al. (2001), ^26^Norman et al. (2004), ^27^Pack et al. (1999), ^28^Paisley et al. (1998), ^29^Petty et al. (2005), ^30^Pittman and Krementz (2016), ^31^Porter et al. (1983), ^32^Pollentier et al. (2014), ^33^Reynolds and Swanson (2010), ^34^Roberts and Porter (1998a), ^35^Roberts and Porter (1998b), ^36^Seiss et al. (1990), ^37^Thogmartin and Johnson (1999), ^38^Tyl et al. (2020), ^39^Vangilder and Kurzejeski (1995), ^40^Wright et al. (1996), ^41^Wood et al. (2019), ^42^Yeldell et al. (2017).

### 3.2 Scoping review‐‐adults

The effects of hunting season timing and duration were the most studied topic for adult survival (*n* = 6; 9%), with most of these studies focusing on males (Table 2). The next most studied topics were related to intrinsic factors, including individual age/body condition (*n* = 5; 7%), reproductive status (*n* = 4; 6%), and space use (*n* = 1; 1%; Table 2). Three studies (4%) evaluated how habitat composition at the landscape scale influenced survival, with only two studies (3%) evaluating the effects of management on survival (both related to supplemental feeding in the winter). Only one study (1%) directly evaluated the effects of weather on adult survival. Similar to nesting studies, no studies (0%) evaluated biotic interactions (e.g., changes in predator communities or disease), despite predation being frequently reported as the main source of adult mortality (Table 2).

### 3.3 Scoping review‐‐poults

Only six studies (8%) reported variables that influenced poult survival, with all these studies using brood flush counts to estimate survival (Table 2), and only one study that quantified poult survival from both marked poults and flush counts (Hubbard et al., 1999b). Three (4%) of those studies evaluated the effects of breeding season weather on poult survival. Two studies (3%) evaluated both landscape‐scale and fine‐scale habitat factors on poult survival, and one study (1%) evaluated the effects of movement and space use on poult survival.

### 3.4 Life‐stage simulation analysis

From the 89 peer‐reviewed papers, we recorded 1144 vital rate estimates from all subspecies of wild turkey and documented 976 vital rates specific to eastern wild turkey (85% of all vital rates reported) from 68 papers. Of those 976 vital rate estimates, 637 (65%) were relevant for our analysis (Appendix 1). Further, 500 (51%) included a usable metric of variation and 174 (18%) provided female estimates appropriately separated by stage class (Table 1, Figure 2).

**FIGURE 3:**
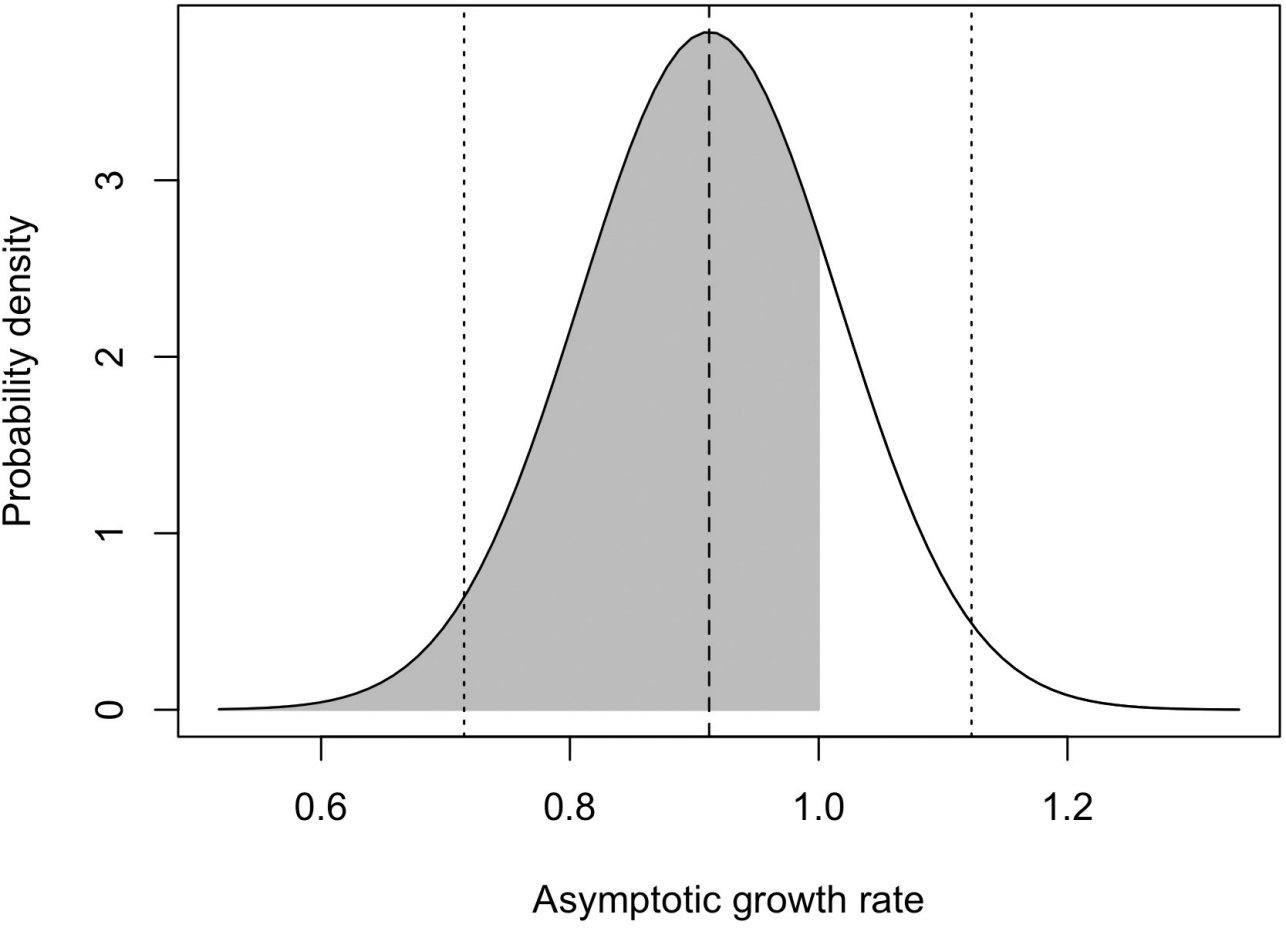
Estimated asymptotic growth rate (*λ*) for eastern wild turkey, 1970–2021. Dashed line represents mean value from 10,000 iterations, and dotted lines represent 95% confidence intervals. Values < 1 indicate declining population size, while values > 1 indicate increasing population size. Shaded area (81%) indicates values representing a declining population (*λ* < 1), and unshaded area shows area indicating an increasing population (*λ* > 1).

Our estimated mean *λ* across 10,000 replicates was 0.91 (95% CI = 0.71, 1.12; Figure 3), representing a mean estimate of 9% annual decline in wild turkey abundance. Of the 10,000 model iterations, 81% of lambda estimates were <1 indicating a declining population trend (Figure 3). The mean elasticities across all replicates were 0.05 for second‐year (SY) adult female reproduction, 0.21 for SY adult female survival, 0.21 for after‐second‐year (ASY) adult female reproduction, and 0.53 for ASY adult female survival, indicating that ASY adult female survival had the greatest proportional effect on population trajectory (Table 3).

**TABLE 3:** Amount of variation in asymptotic growth rate, *λ*, explained by each vital rate for second‐year individuals (SY) and after‐second‐year (ASY) adult eastern wild turkeys, as determined by coefficient of determination (*R* ^2^).

Vital rate	SY	ASY
Incubation initiation (first nest)	0.00	0.01
Apparent nest success (first nest)	0.01	0.05
Clutch size (first nest)	0.00	0.00
Hatching rate	0.00	0.05
Apparent poult survival	0.04	0.51
Incubation initiation (renest)	0.00	0.01
Apparent nest success (renest)	0.00	0.01
Clutch size (renest)	0.00	0.00
Reproduction^a^	0.08	0.74
Annual survival	0.11	0.16

*Note*: Vital rate estimates were derived from studies published between 1970 and 2021.

^a^ Reproduction estimated from Equation (1).

**FIGURE 4:**
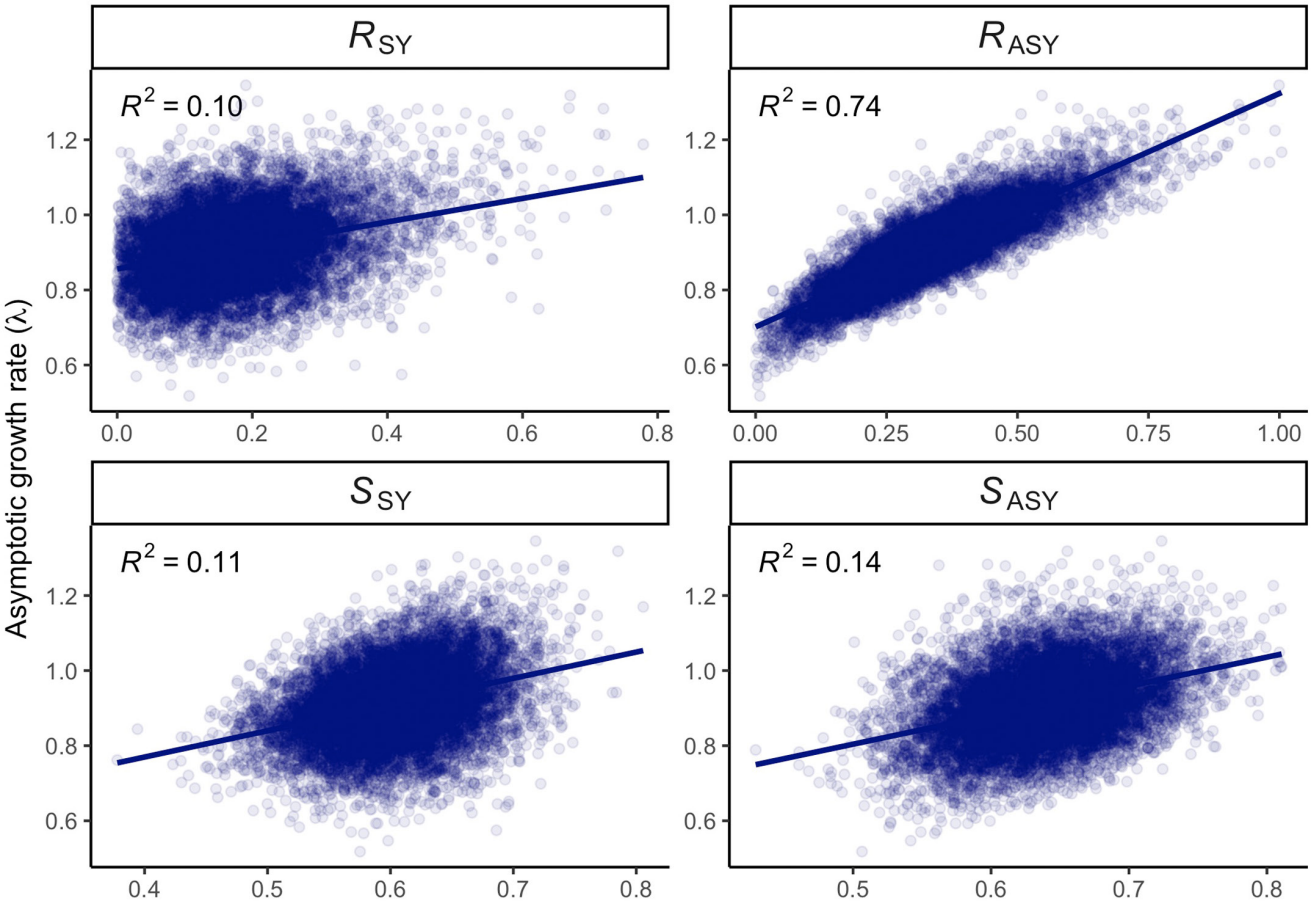
Population growth rate (*λ*) regressed on reproduction (*R*) and survival (*S*) for second‐year individuals (SY) and after‐second‐year adults (ASY) for 10,000 simulated population model replicates generated from our life‐stage simulation analysis. Coefficient of determination (*R* ^2^) values are presented for each vital rate indicating the amount of variation in *λ* explained by each vital rate.

Through our linear regression analysis, we determined that 12% of variation in *λ* was explained by ASY adult female survival and 74% was explained by ASY adult female reproduction (Table 3, Figure 4). Of the component vital rates making up ASY adult female reproduction, by far the most influential was apparent poult survival (explaining 51% of variation in *λ*); each of the other components of ASY adult female reproduction on its own accounted for 7% or less of variation in *λ* (Table 3). Variation in reproduction for second‐year females explained only 8% of the variation in *λ* (Table 3).

## 4 DISCUSSION

Understanding the relative importance of different life history stages for population dynamics and the influence of biotic and abiotic factors on these life stages has been a foundational tenet of wildlife ecology since the earliest stages of the profession (Leopold, 1933). Using an LSA incorporating vital rates published over the last 50 years from the eastern wild turkey's entire historic distribution, our results highlight the importance of ASY adult survival and reproduction for determining population trends. Further, we estimated a mean population trend of 9% decline per year (*λ* = 0.91, 95% CI = 0.71, 1.12), based on the best available information in the wild turkey literature. However, regional monitoring will be critical for clarifying patterns in wild turkey declines throughout their distribution (Chamberlain et al., 2022). The proposed causes for recent declines have included a range of factors, including reduced habitat quality and quantity, changes in predator abundance and predator communities, weather variability, increased disease prevalence, and changes in hunting pressure (Casalena et al., 2015). Our review of the wild turkey literature suggests that there may be substantial gaps in our knowledge of wild turkey demographics and how several of these factors influence vital rates. These gaps may limit managers' ability to adequately address wild turkey declines. For many factors that can influence wild turkeys, our knowledge is sparse (e.g., effects of weather or habitat during certain life stages) or nearly nonexistent (e.g., effects of changing predator communities). Additionally, information regarding certain demographic stages is limited or unavailable (i.e., poult or youth survival), leaving critical gaps in our knowledge of wild turkey demography.

Gamebird survival and reproduction is often the result of complex interactions between factors such as habitat, predator–prey dynamics, weather, and harvest pressure, among other factors (Howell et al., 2021; Powell et al., 2022; Shipley et al., 2020; Tanner et al., 2017). We found most of the focus in the literature has been on evaluating the effects of harvest on adult survival and habitat conditions on nest success, while other sources of variation in wild turkey vital rates have received much less attention. Given the concern of the effects of hunting and the potential influence of habitat conditions on nest success (i.e., vulnerability to nest predators and exposure to adverse weather), it is not surprising the focus on understanding wild turkey population declines has been on hunting and nesting habitat. However, this relatively narrow focus may make assessing the drivers of wild turkey declines difficult. For example, while predation is often cited as the primary source of direct mortality for adult female wild turkeys and wild turkey nests, it is also important to understand how factors such as weather, disease, or habitat may interact to alter an individual's risk of mortality from predators or other causes. Disentangling the roles of these different factors, their interactions, and how density dependence may influence their relative importance can shed considerable light on the drivers that regulate or limit wildlife populations (Martínez‐Padilla et al., 2014; Powell et al., 2022). However, approximately half of studies reviewed provided no analysis of mechanisms that may influence reported vital rates. This suggests a need for more hypothesis‐driven research to understand the factors influencing wild turkey populations.

Like previous population modeling efforts for wild turkeys (Pollentier et al., 2014; Roberts & Porter, 1996; Rolley et al., 1998), survival and reproduction of ASY females were among the most influential vital rates for determining wild turkey population growth. Particularly, survival of ASY females had the greatest elasticity, suggesting minor changes in this parameter can significantly affect population growth; however, this parameter also had comparatively low variation. In contrast to the survival of ASY females, reproduction by this stage class was less elastic, but was much more variable and explained a greater amount of variance in *λ* compared with the survival of this stage class. These relationships suggest wild turkeys may have more of a “survivor” life history strategy (Sæther, 1988), similar to other long‐lived galliform species (Taylor et al., 2012). Notably, the higher variance in ASY reproduction suggests managers may have greater ability to influence this life stage through management compared with ASY survival. However, the actual degree of control managers will have on wild turkey vital rates depends on the abiotic or biotic conditions that limit them. For example, managers may have only limited ability to improve survival or reproduction if weather is the dominant driver but may have greater control if other factors such as habitat quality, predators, or harvest are the primary drivers. Therefore, better quantifying the effects of different factors on a wild turkey vital rates is essential for determining the most appropriate management approaches going forward. For populations where limiting factors are largely unknown, the use of precautionary measures such as eliminating female harvest may be advisable given the importance of adult females to population growth. Finally, while changes in one or a few vital rates may cause local declines, improvements in multiple vital rates may be required to achieve stable growth for a recovering population (Allen et al., 2022).

Our literature review of wild turkey vital rates revealed several basic research needs in terms of demographic data and data considerations that can improve our understanding of wild turkey demographics. This research needs largely echo challenges highlighted for other galliform species (Sandercock et al., 2008; Taylor et al., 2012). First, most vital rates we extracted could not be included in our LSA because of inconsistencies in reporting sample sizes, error estimates, or stage‐specific results (e.g., reproduction or survival of second‐year individuals versus after‐second‐year individuals), and many studies did not include these basic summary statistics associated with their vital rates estimates. Developing a standardized approach to reporting vital rates in peer‐reviewed works can substantially improve the utility of studies for future research, especially as methods for synthesizing data across studies becomes more widespread.

An important caveat to our LSA model was that we only used vital rates for adult females. This was done to simplify the model structure and reduce the number of parameters to estimate in our models. As a result, our model implicelty assumes female survival primary driver of population trends for wild turkeys. However, this assumption would need to be critically evaluated across populations and regions given that males and females are exposed to different morality risks, particularly with regard to harvest (Isabelle et al., 2018). In fact, a potential hypothesis for wild turkey declines in some regions is that overharvest of males may be suppressing populations (Casalena et al., 2015), with several modelling studies showing male harvest can have an important influence on wild turkey population trajectories (Alpizar‐Jara et al., 2001; McGhee et al., 2008; Stevens et al., 2017). Developing wild turkey population models that incorporate information from both sexes is a critical next step as these models can then be used to explore how different harvest regimes and sources of mortality risk can influence population size.

We also identified a critical knowledge gap regarding demographic data for the poult period and youth survival period. Only six estimates from two studies provided sufficient data on poult survival for inclusion in our model (i.e., Shields & Flake, 2006; Tyl et al., 2020), and no studies reported youth survival. As a result, our estimates of vital rate contributions to population growth should be viewed with caution, as these missing life stages are highly influential in other galliform species with similar life history strategies (Sandercock et al., 2008, Taylor et al., 2012). In particular, we likely overestimated survival in the youth period as we used information from SY individuals to estimate survival during the youth period. While historically, collecting data on poult and youth survival was limited by the battery life and size of VHF transmitters, this technology has advanced considerably allowing for the monitoring of chick and early youth survival in related species (Sinnott et al., 2022; Terhune et al., 2020), suggesting that a reevaluation of these methods for monitoring poult survival may be justified in wild turkeys. Further, existing methods for monitoring poult survival, such as flush counts, have been shown to be highly biased if imperfect detection or brood mixing behaviors are not accounted for in survival estimates (Dahlgren, Messmer, & Koons, 2010; Dahlgren, Messmer, Thacker, & Guttery, 2010; Kubečka et al., 2021; Orange et al., 2016). A critical evaluation of new and existing techniques for monitoring these periods should be a high priority so that researchers can make more informed decisions when designing studies on these periods. As an alternative to directly monitoring survival, use of quantitative methods, such as integrated population models or statistical population reconstruction (Ahrestani et al., 2017; Clawson et al., 2022; McConnell et al., 2018), may allow for the estimation of information about these missing life stages or alternative parameters such as recruitment. Use of parameters like recruitment is not perfect substitution for directly monitoring poult or youth survival, as the use of these metrics can mask variation in stage‐specific survival, but these approaches may help fill the gaps in our knowledge of these difficult‐to‐study life stages (McConnell et al., 2018). Finally, the use of sensitivity analysis can help establish the importance of these life stages and provide managers with a baseline to guide management (Davis et al., 2016).

We also had to make several critical simplifying assumptions about wild turkey population dynamics for our LSA approach. We assumed limited (or no) correlation among vital rate parameters and that wild turkeys were predominately affected by density‐independent factors. Many wildlife species exhibit correlations among vital rates either when the same environmental conditions influence multiple life stages (i.e., unfavorable weather reducing poult and adult survival) or when they experience trade‐offs between life history stages where behaviors or energy expenditure in one stage may influence the survival of another life stage (Sæther, 1988). Such trade‐offs have been observed in wild turkey (Byrne & Chamberlain, 2018; Yarnall et al., 2020). Alpizar‐Jara et al. (2001) noted that assuming independence among demographic parameters might be tenuous for wild turkeys but that available data at the time were “far from adequate” to estimate correlations. Twenty years later, the situation has changed little, leading us and others to make the similar simplifying assumption (Lehman et al., 2022). Additionally, recent work has suggested that wild turkey populations may be influenced by density‐dependent factors, with productivity potentially being negatively correlated with population size in eastern wild turkeys (Bond et al., 2012; Byrne et al., 2015). Due to the coarse resolution of the data that these conclusions are based on (i.e., poult: hen counts), it is unclear if or what life stages are affected by density dependence and the potential underlying mechanisms. While density dependence in turkeys is poorly understood at present, assumptions related to the strength of density dependence can significantly affect estimates of population growth in wild turkeys (McGhee et al., 2008). Accounting for correlation structures and potential density dependence is necessary next step for improving wild turkey population models and subsequent management strategies.

## 5 CONCLUSIONS

Based on our LSA, parameterized with the best available published data of wild turkey vital rates, management efforts that focus on increasing the survival and productivity of adult females will likely have the greatest effects on wild turkey population growth. However, in addition to collecting data on poorly studied life history stages, efforts to better quantify how biotic and abiotic factors, such as weather, disease, predators, and habitat and their interactions influence wild turkey mortality and productivity should be a high priority for research, as this is essential for guiding management and improving population models for wild turkeys. This may include using methods that improve our ability to assess the factors that increase mortality risk, relate variation in predator communities to wild turkey demographics, and evaluate changes in vital rates based on habitat management. Past wild turkey research has provided a solid foundation of knowledge regarding the ecology of this species, but as analytical methods for modeling wildlife populations (Gardner et al., 2010; Schaub & Abadi, 2011), technology for monitoring survival and space use of individuals (Collier & Chamberlain, 2011), and tools to monitor disease occurrence (Kunkel et al., 2022; Shea et al., 2022) improve, we believe that wild turkey research and our ability to manage this species can experience rapid advancements through the development of new and targeted research goals. By reviewing and synthesizing the literature on wild turkey vital rates, we highlight patterns in the topics that have been studied and identify gaps in our knowledge base. Research that builds on existing wild turkey ecology research while providing a more mechanistic and hypothesis‐driven understanding of the underlying causes of variation in vital rates is foundational for conserving this species.

## AUTHOR CONTRIBUTIONS

**David W. Londe:** Conceptualization (equal); data curation (lead); formal analysis (equal); investigation (lead); methodology (equal); validation (equal); writing – original draft (lead); writing – review and editing (equal). **Anna K. Moeller:** Conceptualization (equal); formal analysis (lead); investigation (equal); methodology (equal); visualization (equal); writing – original draft (supporting); writing – review and editing (equal). **Paul M. Lukacs:** Formal analysis (supporting); investigation (supporting); methodology (supporting); writing – review and editing (supporting). **Samuel D. Fuhlendorf:** Conceptualization (supporting); writing – review and editing (supporting). **Craig A. Davis:** Conceptualization (supporting); writing – review and editing (supporting). **Robert Dwayne Elmore:** Conceptualization (equal); funding acquisition (equal); methodology (supporting); project administration (supporting); supervision (equal); writing – original draft (supporting); writing – review and editing (equal). **M. Colter Chitwood:** Conceptualization (equal); investigation (equal); methodology (equal); project administration (equal); supervision (equal); writing – original draft (supporting); writing – review and editing (equal).

## FUNDING INFORMATION

This work is supported by Hatch OKLO3056 project from the USDA National Institute of Food and Agriculture and the Oklahoma Agricultural Experiment Station at Oklahoma State University.

### OPEN RESEARCH BADGES

This article has earned an Open Materials badge for making publicly available the components of the research methodology needed to reproduce the reported procedure and analysis. All materials are available at https://doi.org/10.6084/m9.figshare.21968603.v1.

## Data Availability

All vital rates recorded from the literature and used in our analysis can be found at https://doi.org/10.6084/m9.figshare.21968603.v1.

## APPENDIX 1 Definitions of vital rates used in life‐stage simulation analysis

We populated our life‐stage simulation analysis (LSA) with turkey vital rates derived from the literature. Here, we provide additional information on how each vital rate was defined and estimated by these studies.

- Annual Survival—Survival for second‐year adults (*S*_SY_) and after‐second‐year adults (*S*_ASY_) was estimated as the proportion of individuals in that age class in year *t* that survived to the next year (*t* + 1). In most studies, survival is estimated from individuals marked with VHF or GPS radio tags that allow individuals to be monitored throughout the annual cycle. Survival was estimated as the percent of individuals that survived over a given period or by extrapolating daily survival estimates over the period of interest.
- Incubation initiation—We used the term incubation initiation (II) to refer to the vital rate called nest initiation (NI) in published literature to emphasize the fact that observations of this vital rate occur when hens begin to incubate nests, not when they begin to build nests or lay eggs. Typical estimates of nest initiation likely underestimate the actual proportion of females that attempted a nest, as many nests that are lost prior to incubation are not detected. However, it should be noted that estimates of nest success, clutch size, hatching rate, and poult survival are also conditional on incubation (not nest initiation), so this did not bias our estimates of reproduction or LSA results. Incubation initiation rate for first nests (II_1_) was defined as the proportion of females who were alive at the beginning of the nesting period that attempted at least one nest. Because turkeys only attempt to renest if the first nest fails, incubation initiation rate for second nests (II_2_) was conditional on failure of the first nest. II_2_ was defined as the proportion of females with a failed first nest who then attempted a second nest. While an individual female may attempt as many as three to four nests, these are rare, and we did not include third or fourth nesting attempts in our model. We report incubation initiation separately for second‐year adults (II_1,SY_ and II_2,SY_) and after‐second‐year adults (II_1,ASY_ and II_2,ASY_).
- Nest success—Nest success for first and second nests (NS_1_ and NS_2_) was estimated as the proportion of nests that survived from detection to the end of the incubation period (approximately 25–28 days) and produced ≥1 poult. Nest success estimates from most studies typically overestimate actual nest success because they do not account for nests lost prior to detection. As a result, reproductive estimates may be biased high in some cases. We reported nest success separately for second‐year adults (NS_SY_) and after‐second‐year adults (NS_ASY_).
- Clutch size—Clutch size for first nests (*C*_1_) and renests (*C*_2_) was recorded as the average number of eggs laid per nest. We assumed an average of 1:1 sex ratio in each clutch and multiplied clutch sizes by 0.5 to create an estimate of the approximate number of female eggs per clutch. We reported clutch size separately for second‐year adults (*C*_SY_) and after‐second‐year adults (*C*_ASY_).
- Hatching rate—Hatching rate for first nests (*H*_1_) and renests (*H*_2_) is defined as the proportion of eggs in a nest that hatched. We reported clutch size separately for second‐year adults (*H*_SY_) and after‐second‐year adults (*H*_ASY_). Hatching rate is typically estimated as the number of hatched eggs in a successful nest (produced ≥1 poult) divided by the number of eggs in the nest at the start of the incubation period.
- Poult survival—We defined poult survival (PS) as the proportion of poults surviving from hatch to 28 days. We selected 28 days as our cutoff for poult survival because most studies only report survival until 4–5 weeks following hatch and because while poults may still be associated with females at this age, they are also largely independent of the female, making their own decisions about foraging and roosting. We only used poult survival estimates that were reported separately for second‐year adults (PS_SY_) and after‐second‐year adults (PS_ASY_). Most studies relied on simple flush counts of poults at approximately 28 days and estimated survival as the proportion of chicks observed during a flush count relative to the number of eggs that hatched in a given female's nest. These methods are likely biased in that they do not account for brood amalgamation or adoption of lost poults by unmarked females. These methods also assume complete detection of poults during the flush count which is an assumption that is largely unverified in turkeys but has been shown to be untrue in other galliforms.
- Youth Survival—No studies reported survival estimates for the period between poult fledging and an individual's first breeding season, which roughly corresponds to the ages 28 days to 365 days. We estimated this youth survival (*S*_Y_) following a procedure similar to Roberts and Porter (1996). To do this, we assumed daily survival of individuals during this period would be equal to daily survival rates of second‐year adults. We calculated youth daily survival as *S*_SY_^(1/365)^, and then we calculated survival over the period of 28–365 days by raising daily survival to the power of (365–28).
